# Down-Regulation of Deacetylase HDAC6 Inhibits the Melanoma Cell Line A375.S2 Growth through ROS-Dependent Mitochondrial Pathway

**DOI:** 10.1371/journal.pone.0121247

**Published:** 2015-03-16

**Authors:** Jun Bai, Yun Lei, Gai-li An, Li He

**Affiliations:** Department of Medical Oncology, Shaanxi Provincial People's Hospital, The Third affiliated Hospital of the School of Medicine Xi'an JiaoTong University, Xi’an, P. R. China; Rutgers University, UNITED STATES

## Abstract

Previous studies have shown that histone deacetylase 6 (HDAC6) plays critical roles in many cellular processes related to cancer. However, its biological roles in the development of melanoma remain unexplored. Our aim was to investigate whether HDAC6 has a biological role in human melanoma development and to understand its underlying mechanism. In the present study, HDAC6 expression was up-regulated in melanoma tissues and cell lines. Knockdown of HDAC6 significantly inhibited the proliferation and colony formation ability of A375.S2 cells, promoted cell arrest at G0/G1 phase and apoptosis. Additionally, western blotting assay showed that HDAC6 silencing suppressed Bcl-2 level and enhanced Bax level, then activated caspase-9 and caspase-3, and further activated the release of cytochrome c from mitochondria to cytoplasm, finally induced cell apoptosis involving the mitochondrial pathway. Knockdown of HDAC6 triggered a significant generation of ROS and disruption of mitochondrial membrane potential (MMP). Furthermore, ROS inhibitor, NAC reduced HDAC6 siRNA-induced ROS production, and blocked HDAC6 siRNA-induced loss of MMP and apoptosis. NAC also significantly blocked HDAC6 siRNA-induced mtDNA copy number decrease and mitochondrial biogenesis and degradation imbalance. In conclusion, the results showed that knockdown of HDAC6 induced apoptosis in human melanoma A375.S2 cells through a ROS-dependent mitochondrial pathway.

## Introduction

Histone deacetylase 6 (HDAC6), a special class IIb histone deacetylase, is located on the Xp11.23 chromosome [1]. HDAC6, which is predominantly in the cytoplasm, is a unique member of class II because it contains two homologous, catalytic domains that are fully functional [2]. HDAC6 plays an important role in many cellular processes related to cancer, including the cell stress response, cell migration and motility and cancer-related signaling pathways. HDAC6 has been found in the brain, breast, colon, ovary, pancreas, prostate and heart and may be up-regulated in the brain, breast, ovary and pancreas cancers [3]. The expression of HDAC6 in diverse tumours suggests an important role of HDAC6 in cancer.

Mitochondria perform energy production and metabolism to maintain the cellular homeostasis and they are the most important sensors for apoptosis [4]. Mitochondria are the major sites for ROS production, and excessive generation of ROS results in cells injury and death [5]. ROS are not only byproducts of mitochondrial respiration, but also key signaling molecules that regulate mitochondrial dysfunction [6,7]. Mitochondria manage apoptotic signals that include changing of electron transport, loss of mitochondrial membrane potential (MMP), generation of ROS and release of caspase activators [8]. A breakdown in the MMP is an invariant feature of early apoptosis [9]. Down-regulation of HDAC6 causes a reduction in the mitochondrial enzymes activity, indicating that HDAC6 regulates mitochondrial metabolism [10]. Therefore, targeting HDAC6 for cancer therapy may be a good strategy due to its important role in providing an advantage to cancer cells to survive [11].

In recent decades, the incidence of melanoma has dramatically increased, thus, understanding melanoma at the molecular level and identifying its novel molecular targets are needed to improve therapeutic strategies. Therefore, the purpose of this study was to observe the effect of HDAC6 in human malignant melanoma cell and to characterize the underlying molecular mechanisms via the ROS-mediated apoptosis by observing a series of cellular apoptotic pathways including mitochondrial function.

## Materials and Methods

### Tissue samples

From February 2009 to December 2012, 23 melanoma tissues and 23 distant normal dermatic tissues were obtained from patients (age: 47.35 ± 4.05 years and 58 ± 8.32 years) who were admitted to the Department of Medical Oncology, Shaanxi Provincial People's Hospital. This study was conducted according to the guidelines in the Declaration of Helsinki and all procedures involving human subjects were approved by the Human Ethics Committee of Shaanxi Provincial People's Hospital and Xi’an Jiaotong University, PR China. Written informed consent was obtained from all participants.

### Cells and cell culture

The human melanoma cell lines A375.S2, SK-MEL-28 and HT-144 and the human immortalised keratinocytes (HaCaT) and normal human epidermal melanocytes (PIG1), were purchased from American Type Culture Collection (Manassas, VA, USA) and were maintained in RPMI-1640 or DMEM or 254 supplemented with 10% fetal bovine serum, 100 U/ml penicillin G and 100 μg/ml streptomycin sulphate or with human melanocyte growth supplement in a 5% CO_2_-humidified atmosphere at 37°C.

### siRNA, RNA extraction and real-time analysis

Two melanoma cell lines, i.e., A375.S2 and SK-MEL-28 were used to detect HDAC6 expression. The cells were seeded on 6-well plates to 40–50% confluency and then transfected with non-targeting siRNA or siRNA directed against human HDAC6 (Santa Cruz Biotech, Santa Cruz, CA, USA) using Lipofectamine 2000 (Invitrogen, Carlsbad, CA, USA) according to the instructions provided by the manufacturer. The effect of siRNA treatment on expression of HDAC6 was determined by quantitative real-time PCR and western blot 48 or 72 h post-transfection.

Total RNA was isolated from cells using TRIzol reagent, and reverse transcriptions were performed using the Takara RNA PCR kit (Takara, Dalian, China) following the manufacturer’s instructions. Quantitative PCR was performed using a SYBR Green Premix Ex Taq (Takara) on a real-time PCR system (Eppendorf, Germany). The primer sequences were used as previously described [12]. The human β-actin gene and GAPDH gene served as the endogenous reference genes. The relative expression ratio was calculated by the 2^-ΔΔCT^ method.

### Cell proliferation assay

Cell growth was analyzed using a Cell Counting Kit-8 (CCK-8) (Beyotime, Jiangsu, China) as previously reported. Briefly, cells suspended in DMEM medium containing 10% fetal bovine serum were seeded in 96-well plates at a concentration of 2000 cells per well and incubated for 24, 48 and 72 h. CCK-8 solution was added to each well, and the cultures were incubated at 37°C for 60 min. Absorbance at 450 nm was measured using a microplate reader (SPECTRAmax PLUS384, Molecular Devices, USA). Experiment was done in triplicate and average OD450 was used to calculate the growth inhibition rate.

### Clone formation assay

Cells were seeded at 1.0 × 10^4^ cells/well in a 60 mm culture dish and left to form clones for two weeks. Cultures were stained with 0.1% crystal violet, and the number of clones within a 2 × 2 cm grid (on the culture plates) was scored to determine the clone-forming ability of the cells. Clones containing over 50 cells were counted.

### Cell cycle analysis

After the indicated treatments, cells were trypsinised, washed with PBS, fixed with 70% ethanol at 4°C overnight and treated with RNaseA in the dark at room temperature for 30 min. The cells were then resuspended in 0.05 mg/ml propidium iodide (PI) and analyzed by flow cytometry on a FACSCalibur apparatus (BD Biosciences, San Jose, CA, USA).

### Cell apoptosis assay

PI and Annexin V-FITC were used for the determination of cell apoptosis. Briefly, the cells transfected with HDAC6-siRNA or NC-siRNA for 24 h were trypsinized, resuspended in AnnexinV-binding buffer and incubated with AnnexinV-FITC/PI in dark for 20 min. The samples were detected using a FACS Calibur flow cytometer (BD Biosciences) and analyzed by FloMax software.

### Senescence-associated β-galactosidase (SA-β-gal) staining

Senescence-associated β-galactosidase was performed with a senescence-associated β-Galactosidase Staining Kit (Beyotime) according to the manufacturer’s instructions. Briefly, cells were washed and fixed with 4% paraformaldehyde for 15 min at room temperature. Next, the cells were incubated overnight at 37°C in darkness with 0.05 mg/ml X-gal.

### ROS generation assay

Changes in the intracellular concentration of ROS were detected using dichlorodihydrofluorescein diacetate (H_2_DCF-DA). The cells transfected with HDAC6-siRNA or NC-siRNA for 24 h were washed twice with PBS and incubated with DCFH-DA at 37°C for 50 min in the dark. Fluorescent signals were then observed using a fluorescence spectrometer at 485 nm (excitation) and 538 nm (emission). The intracellular ROS concentration was quantified as the relative DCF fluorescence per sample protein amount (BCA method).

### Mitochondrial membrane potential (MMP) assay

MMP was assessed using the lipophilic cationic probe 5,5′,6,6′-tetrachloro-1,1′,3,3′-tetraethylbenzimidazolylcarbocyanine iodide (JC-1) (Invitrogen) for quantitative fluorescence measurements according to manufacturer’s instructions.

### Mitochondrial DNA (mtDNA) content assay

mtDNA was assessed as described previously [13]. Briefly, total DNA was extracted and quantitative PCR was done using 18S rRNA primers for a nuclear target sequence and primers for mitochondrial DNA target using mitochondrial D-loop. The following primers were used: mitochondrial D-loop forward, 5’-CACCCAAGAACAGGGTTTGT-3’, reverse, 5’-TGGCCATGGGTATGTTGTTAA-3’; 18S rRNA forward: 5’-TAGAGGGACAAGTGGCGTTC-3’, reverse, 5’-CGCTGAGCCAGTCAGTGT-3’. mtDNA content was determined as the ratio of mitochondrial D-loop to 18S rRNA. Final results are presented as percentage of control.

### Mitochondrion isolation

Preparation of mitochondrial and cytosolic proteins was performed using the mitochondria/cytosol fractionation kit according to the manufacturer's protocol (Beyotime, China). Briefly, cells were resuspended in a hypotonic buffer for 10 min. The heavy membrane fraction was sedimentated at 11,000 g for 10 min after removal of nuclei and unbroken cells. The supernatant was further centrifuged at 12,000 g for 10 min. The supernatants of the 12,000 g spin were used as the cytosolic fraction. The mitochondria and cytosolic fraction were then subjected to western blot.

### Western blot analysis

Cells samples transfected with HDAC6-siRNA or NC-siRNA for 48 h were harvested by trypsin and lysed with Western and IP lysis buffer (Beyotime). The lysates were homogenised, and protein concentrations were determined using the BCA Protein Assay kit (Pierce, Rockford, IL, USA). After immunoblotting, the membranes were blocked for 1 h in 5% (w/v) milk/TBST with 5% non-fat milk. Antibodies directed against HDAC6, cytochrome c, cleaved caspase-3, cleaved caspase-9, Bax, Bcl-2, PPAR-coactivator-1alpha (PGC-1α), Mfn 2, DRP1, COX-IV, GAPDH and β-actin were all purchased from Santa Cruz Biotechnology (Santa Cruz), and chemiluminescent detection was performed using an ECL detection kit (Pierce). The results were analyzed using the Quantity One software to obtain the optical density ratio of the target protein to β-actin.

### Statistical analysis

Results were expressed as the mean ± SEM (standard deviation) of more than three independent experiments. Statistical significance was calculated by Student’s t-test between the two groups or one-way analysis of variance (ANOVA) and P value for a multiple comparison test. The level of statistical significance was defined as *P values < 0.05.

## Results

### The high level of HDAC6 expression in melanoma tissues and cell lines

**Fig 1:**
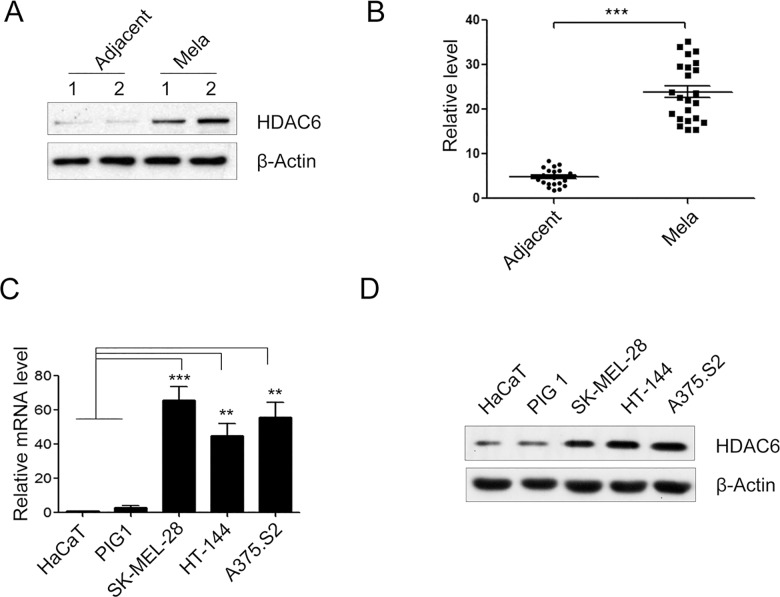
The expression of HDAC6 was up-regulated in melanoma tissues and cell lines. (**A**) Representative melanoma tissue (Mela) and adjacent normal dermatic tissue (Adjacent) samples of HDAC6 protein expression were determined by Western blot. β-actin was used as internal loading controls for the cell lysates in the Western blot analysis. (n = 23). (**B**) The protein expression of HDAC6 in 23 pairs of melanoma tissue (Mela) and adjacent normal dermatic tissue (Adjacent) samples. (**C**, **D**) The mRNA level and protein expression of HDAC6 were analyzed in several melanoma cell lines, A375.S2, SK-MEL-28, HT-144 and human immortalised keratinocytes (HaCaT) and normal human epidermal melanocytes (PIG1) by quantitative real time PCR (qRT-PCR) (**C**) and Western blot (**D**). (n = 4). Bars represent the mean ± S.E.M. values. Statistical significance (**P < 0.01, ***P < 0.001).

HDAC6 expression in human malignant melanoma (n = 23) and its adjacent, normal cancer dermatic tissue (n = 23) was assayed by Western blot. We found that HDAC6 protein expression was significantly up-regulated in malignant melanoma tissue, whereas weak expression was found in the adjacent, normal cancer dermatic tissue (Fig. 1A and B, ***P < 0.001). We also analyzed HDAC6 expression in several melanoma cell lines by quantitative real time PCR (qRT-PCR) and Western blot. The higher expression of HDAC6 in three melanoma cell lines (A375.S2, SK-MEL-28 and HT-144), compared with a normal skin epithelium cell line (HaCaT) or compared with normal human epidermal melanocytes (PIG1), was examined (Fig. 1C and D, **P < 0.01 and ***P < 0.001, respectively). Therefore, our results demonstrated that HDAC6 expression was up-regulated in melanoma tissues and cell lines.

### Effect of HDAC6 siRNAs on the expression level of HDAC6 in A375.S2 cells

**Fig 2:**
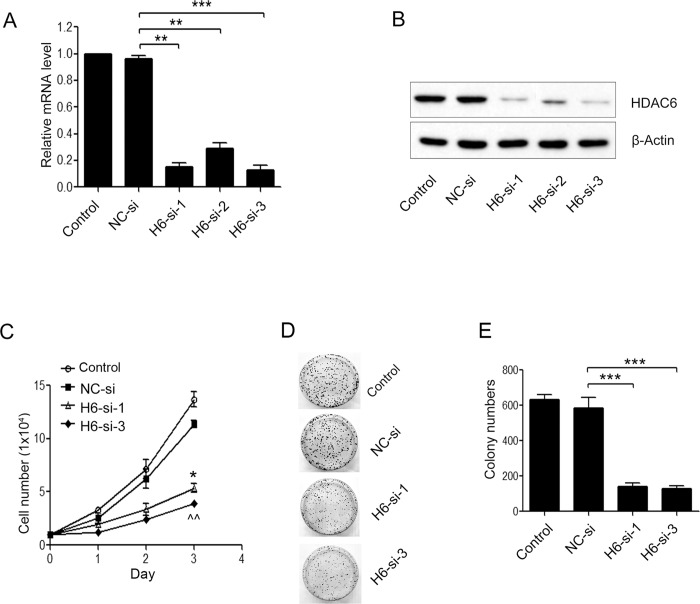
HDAC6 silencing inhibited A375.S2 cell proliferation and colony formation. (**A**, **B**) Selective down-regulation of HDAC6 expression in A375.S2 cells that were treated for 24 or 48 h with the negative control (NC-si) or targeting-HDAC6 (H-6-si-1, -2 and-3) siRNAs or cells alone. The mRNA and protein levels of HDAC6 were determined by qRT-PCR analysis (**A**) and Western blot (**B**). (**C**) A Cell Counting Kit-8 (CCK-8) assay determined the growth condition of H6-si-1 or-3 treated and NC-si-treated A375.S2 cells. (**D**) Colony formation and (**E**) quantification of colony number. Clonogenic assay showing the effect of a two weeks incubation with H6-si-1 or-3 on the formation of cell colonies compared with NC-si-transfected cells. Bars represent the mean ± S.E.M. values. (n = 4). Statistical significance (*P < 0.05, **P < 0.01, ^^*P* < 0.01, ***P < 0.001). NC-si, NC-siRNA treated group; H6-si-1, HDAC6-siRNA-1 treated group; H6-si-3, HDAC6-siRNA-3 treated group.

Small interfering RNA (siRNA) therapies for cancer have begun clinical development [14]. Thus, the effects of siRNA-mediated knockdown of HDAC6 were investigated in malignant melanoma. A panel of HDAC6 siRNAs was respectively transfected and the extent of HDAC6 protein expression was measured after HDAC6-siRNAs transfection over a 48-h incubation period. Quantitative PCR analysis showed that HDAC6 siRNA-1 and-3 infection resulted in the lower mRNA levels in A375.S2 cells compared with negative control (NC) siRNA-treated A375.S2 cells (Fig. 2A, **P < 0.01 and ***P < 0.001, respectively). Western blot analysis showed that the HDAC6 protein was significantly reduced in HDAC6 siRNA-1 and-3 A375.S2 cells (Fig. 2B), consistent with its mRNA reduction. Taken together, these data suggested that HDAC6 siRNA could significantly suppress the endogenous HDAC6 expression.

### Knockdown of HDAC6 inhibited cell proliferation and colony formation

Cell proliferation was examined using Cell Counting kit-8 at the time points of 24, 48 and 72 h. The average optical density of the HDAC6 siRNA-1 and-3-treated A375.S2 cells were all significantly lower than that of the NC siRNA-treated A375.S2 cells on day 3 (Fig. 2C. *P < 0.05 or ^^*P* < 0.01 for the NC siRNA group), indicating that HDAC6 knockdown inhibited A375.S2 cell growth. In addition, the colony formation assay displayed a dramatic decrease of 3-fold in colony number when A375.S2 cells were treated with the HDAC6 siRNA-1 or-3 relative to the NC siRNA-treated A375.S2 cells (Fig. 2D and E, ***P < 0.001 for the NC siRNA group). Thereafter, HDAC6 siRNA-1 and-3 were used for all subsequent experiments.

### Knockdown of HDAC6 induced cell arrest in the G0/G1 phase and apoptosis, but not affected senescence

**Fig 3:**
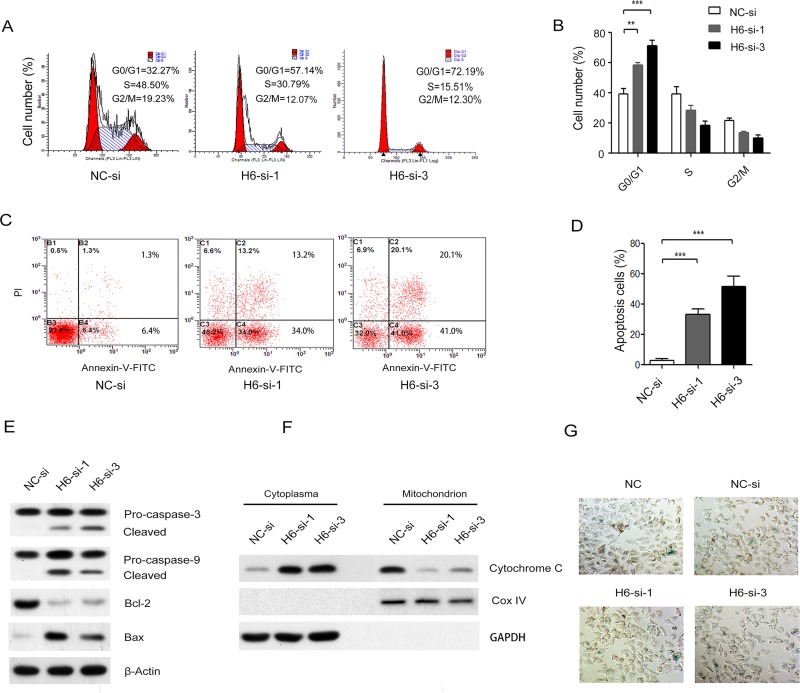
HDAC6 down-regulation arrested cell cycle and induced apoptosis, but not affected senescence. (**A**) FACS analysis of NC-si-treated and H6-si-1 or-3-treated A375.S2 cell cycle. (**B**) The cell percentage at different phases was indicated. (**C**) Flow cytometric analysis of apoptosis following staining with PI/AnnexinV-FITC in H6-si-1 or-3-treated and NC-si-treated A375.S2 cells. (**D**) The percentage of PI positive and Annexin V positive cells was indicated. (**E**) The protein levels of caspase-3 and-9, Bax and Bcl-2 expressions were analyzed by Western blot in H6-si-1 or-3-treated and NC-si-treated A375.S2 cells. (**F**) Immunoblotting for cytochrome c using cytosolic and mitochondrial fractions. GAPDH or Cox IV antibody was used to normalize for protein loading. (**G**) Senescence was observed by the detection of SA-β-Gal-positive cells. Bars represent the mean ± S.E.M. values. (n = 4). Statistical significance (**P < 0.01, ***P < 0.001). NC-si, NC-siRNA treated group; H6-si-1, HDAC6-siRNA-1 treated group; H6-si-3, HDAC6-siRNA-3 treated group.

To investigate the mechanisms leading to cell growth inhibition in HDAC6 siRNA-treated A375.S2 cells, we tested whether HDAC6 knockdown affects cell cycle arrest and apoptosis. HDAC6-siRNA-treated A375.S2 cells arrested cells in G0/G1. The G0/G1 phase cell population was increased from 32.27 to 57.14 and 72.19%, accompanied by the decrease of cells in S phase from 48.5 to 30.79 and 15.51% (Fig. 3A and B, **P < 0.01, ***P < 0.001). Hence, these findings suggest that HDAC6 silencing could regulate cell cycle progression and induced G0/G1 phase arrest in A375.S2 cells.

We then examined the impact of HDAC6 knockdown on cell apoptosis using the stain of AnnexinV-FITC/PI. It was observed that apoptosis increased markedly in HDAC6-siRNA-1 and-3 A375.S2 cells compared with the NC-siRNA group (Fig. 3C and D, ***P < 0.001). Because the bcl-2 family proteins regulate cell apoptosis by functioning as either promoters or inhibitors, levels of bcl-2 family protein were examined. Knockdown of HDAC6 decreased accumulation of Bcl-2 protein expression, but increased expression of Bax protein, then activated caspase-9 and caspase-3 (Fig. 3E), and further activated the release of cytochrome c from mitochondria to cytoplasm by Western blot (Fig. 3F).

Senescence is characterized by molecular and morphological cell changes such as an irreversible cell cycle arrest, an increase of cell size and lysosome [15]. Senescence was observed by the detection of SA-β-Gal-positive cells. The SA-β-Gal-positive cells showed no significant difference between HDAC6 knockdown cells and NC siRNA-treated A375.S2 cells or control A375.S2 cells (Fig. 3G).

### HDAC6 silencing resulted in ROS generation and MMP loss in A375.S2 cells

**Fig 4:**
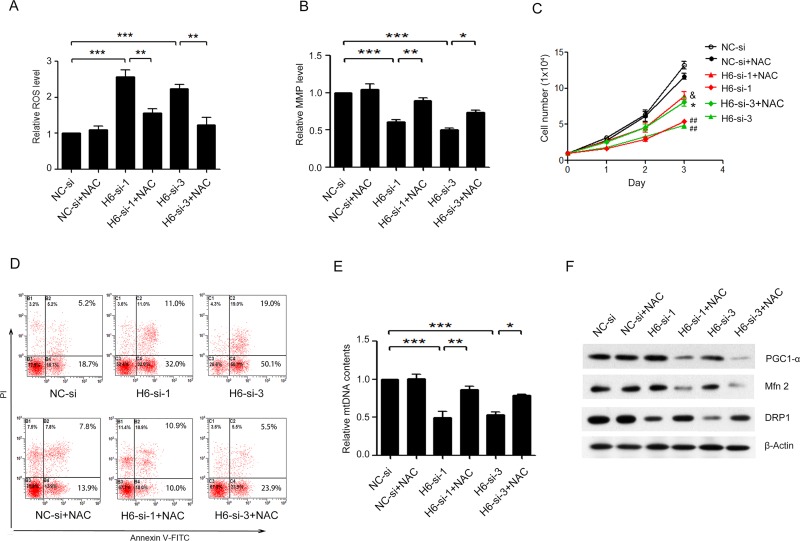
NAC blocked HDAC6 siRNA-induced apoptosis and mitochondrial dysfunction in A375.S2 cells. (**A**) ROS generation and (**B**) mitochondrial membrane potential (MMP) level were detected using DCFH2 and JC-1 staining respectively in H6-si-1 or-3-treated and NC-si-treated A375.S2 cells after pretreatment with or not with 5 mM NAC for 60 min. (**C**) The growth condition of H6-si-1 or-3 treated A375.S2 cells after pretreatment with or not with NAC (^##^ *P* < 0.01, compared to NC-si group; *P < 0.05, compared to H6-si-3 group, ^&^ *P* < 0.05, compared to H6-si-1 group). (**D**) The cell apoptosis was assayed by flow cytometry using PI and Annexin V-FITC double staining. (**E**) Quantitative mtDNA copy number was calculated for D-loop/18S rRNA ratio. (**F**) The mitochondrial biogenesis protein PPAR-coactivator-1alpha (PGC-1α) and the mitochondrial dynamics-related proteins Mfn2 and DRP1 were detected by Western blot. Bars represent the mean ± S.E.M. values. (n = 4). Statistical significance (*P < 0.05, **P < 0.01, ***P < 0.001). NC-si, NC-siRNA treated group; H6-si-1, HDAC6-siRNA-1 treated group; H6-si-3, HDAC6-siRNA-3 treated group.

We further investigated whether HDAC6-siRNA could regulate ROS generation in A375.S2 cells, and the result showed that, in HDAC6-siRNA-1 or-3 A375.S2 cells, ROS generation was increased significantly (Fig. 4A, ***P < 0.001). BITC-induced ROS generation involves the disruption of the MMP in A375.S2 cells [16]. Then, we speculated MMP may be also changed in our study. As shown in Fig. 4B, MMP decreased significantly in HDAC6-knockdown cells compared with NC siRNA-treated A375.S2 cells (Fig. 4B, ***P < 0.001).

### NAC pretreatment blocked HDAC6 siRNA-induced apoptosis and mitochondrial dysfunction in A375.S2 cells

To further examine the role of ROS production in HDAC6 siRNA-induced cell proliferation and apoptosis in A375.S2 cells, cells were pretreated with N-acetyl-L-cysteine (NAC), a ROS scavenger antioxidant. When cells were treated with 5 mM NAC for 60 min after HDAC6-siRNA-1 or-3 transfected for 24 h, ROS level was significantly inhibited (Fig. 4A, **P < 0.01). NAC pretreatment in HDAC6-siRNA-1 or-3 A375.S2 cells inhibited the effects of HDAC6 knockdown including a prevention of MMP loss (Fig. 4B, *P < 0.05, **P < 0.01), an increasing of cell proliferation (Fig. 4C, ^##^ *P* < 0.01, compared to NC siRNA group; *P < 0.05, compared to HDAC6-siRNA-3 group, ^&^ *P* < 0.05, compared to HDAC6-siRNA-1 group) and an inhibition of cell apoptosis (Fig. 4D).

The mtDNA content was quantified by real-time PCR and measured as the ratio of D-loop to 18s rRNA levels. As shown in Fig. 4E, knockdown of HDAC6 resulted in a decrease in the ratio of mitochondrial D-loop/18s rRNA. NAC pretreatment rescued the decreasing of the mtDNA copy number (Fig. 4E, ***P < 0.001, compared to NC siRNA group; **P < 0.01, compared to HDAC6-siRNA-1 group, *P < 0.05, compared to HDAC6-siRNA-3 group).

PGC1-α is the master regulator of mitochondrial biogenesis [17]. The mitochondrial fusion and fission are important processes that result in a continuous remodeling of mitochondrial network dynamics [18]. In our study, we also found that knockdown of HDAC6 decreased biogenesis-related protein PGC1-α, increased mitochondrial fusion-related proteins Mfn2 and decreased the mitochondrial fission-related protein DRP1 expressions (Fig. 4F). But NAC pretreatment strongly inhibited the effects of HDAC6 knockdown on the expression of these proteins (Fig. 4F).

## Discussion

In present study, we reported that HDAC6 expression was up-regulated in melanoma tissues and several melanoma cell lines (Fig. 1), suggesting that this protein contributes to the highly active metabolism of melanoma. It has been demonstrated that RNAi-based therapeutics are effective for the treatment of cancer. Therefore, we demonstrated the potential of silencing HDAC6 expression in melanoma cells would be expected to predominantly be an anticancer therapeutic agent.

In the therapies of carcinoma, promoting the apoptosis of tumor cells is a main approach. Our results revealed that HDAC6 siRNA significantly inhibited melanoma cell proliferation (Fig. 2C) and clone formation abilities (Fig. 2D and E), and arrested cell cycle in G0/G1 phase (Fig. 3A and B). Cell cycle arrest may partially explain the induction of cell growth inhibition of HDAC6 knockdown in melanoma cell line A375.S2. As known in apoptotic cells, phosphatidylserine is translocated from the inner to the outer leaflet of the plasma membrane. The stain of annexinV-FITC/PI was used to explore whether apoptosis occurred. In compared with NC siRNA cells, fluorescence intensities of cells were significantly increased, indicating that silencing of HDAC6 could initiate apoptosis of human melanoma A375.S2 cells (Fig. 3C and D). But senescence results showed no significant difference between HDAC6 knockdown cells and NC siRNA-treated A375.S2 cells or control A375.S2 cells (Fig. 3G).

Mitochondrial plays a key role in the progress of caspase-denpendent apoptosis [19]. The mitochondrial pathway is dependent on the release of cytochrome c from mitochondria into the cytoplasma [20]. The interaction of mitochondria with Bcl-2 family proteins initiates the mitochondrial pathway [21]. Therefore, Bcl-2 family proteins and cytochrome c are considered apoptotic regulators that represent a vital checkpoint within apoptotic pathways [22]. HDAC6 siRNA treatment up-regulated pro-apoptotic member Bax, and down-regulated the anti-apoptotic member Bcl-2 expression (Fig. 3E). The intracellular high Bax/Bcl-2 ratio may contribute to reduce MMP resulting in the release of cytochrome c from mitochondria to cytoplasm matrix. As we know, cytochrome c binds Apaf-1 and procaspase-9, procaspase-9 is cleaved and activates procaspase-3 [23]. As shown in our results, knockdown of HDAC6 activated caspase-9 and-3 (Fig. 3E) and the release of cytochrome c from mitochondria to cytoplasm matrix (Fig. 3F) in A375.S2 cells.

ROS can induce apoptosis in many different cell systems [24–26]. BITC-induced apoptosis via ROS-modulated mitochondria pathways in A375.S2 cells [16]. ROS from mitochondrial may oxidize membrane proteins of mitochondria, change mitochondrial outer membrane permeabilization, and lead to disruption of MMP, which contribute to the release of cytochrome c and apoptosis [27]. In the present study, knockdown of HDAC6 could cause ROS generation (Fig. 4A), and lead to mitochondrial dysfunction including MMP loss (Fig. 4B), mtDNA copy number reduction (Fig. 4E) and unbalanced mitochondrial homeostasis (Fig. 4F). Meanwhile, NAC pretreatment could rescue HDAC6 knockdown-induced MMP loss (Fig. 4B), cell proliferation inhibition (Fig. 4C), cell apoptosis (Fig. 4D) and mitochondrial homeostasis imbalance (Fig. 4F) in A375.S2 cells. Therefore, our results suggested that the main cause of HDAC6 knockdown-induced melanoma cell apoptosis was ROS.

Overall, this study showed that HDAC6 siRNA induced apoptosis of A375.S2 cells through a ROS-mediated mitochondrial pathway. Considering the high expression level of HDAC6 in melanoma tissue and cells and the specificity of HDAC6-siRNA for the inhibition of melanoma cell, silencing HDAC6 expression may be considered as a novel therapeutic approach to treat melanoma. Our results might provide the basis data for gaining a clear understanding of the underlying mechanisms by which it is involved in melanoma.
